# Nomogram for predicting survival in T1–T2 stage patients with supraglottic squamous cell carcinoma

**DOI:** 10.1007/s12672-024-01015-y

**Published:** 2024-05-08

**Authors:** Mulading Maimaitituerxun, Paiheriding Kamilijiang, Aierpati Maimaiti, Yalikun Yasheng, Jun Yong, Ayiheng Qukuerhan, Muredili Mutalifu, Pilidong Kuyaxi, Muzhapaier Mierzhakemu, Aierpati Aierken, Jiulalai Jueraiti, Nilipaer Alimu

**Affiliations:** 1 Department of Otorhinolaryngology, Juntendo University Graduate School of Medicine, 2-1-1 Hongo, Bunkyo-Ku, Tokyo, 113-8421 Japan; 2 Department of Critical Care Medicine, The First Affiliated Hospital of Xinjiang Medical University, Urumqi, 830054 Xinjiang China; 3 Department of Neurosurgery, Neurosurgery Centre, The First Affiliated Hospital of Xinjiang Medical University, Urumqi, 830054 Xinjiang China; 4 Department of Otolaryngology, The First Affiliated Hospital of Xinjiang Medical University, 137 Liyushan South Road, Urumqi, 830054 Xinjiang China

**Keywords:** Supraglottic squamous cell carcinoma (SGSCC), Prognostic factors, Nomogram, SEER

## Abstract

**Background:**

Supraglottic squamous cell carcinoma (SGSCC) is characterized by low differentiation, rapid growth, and inconspicuous initial manifestations. Early detection and prompt treatment can significantly improve survival rates. The main focus of treatment is to maintain optimal laryngeal function.

**Methods:**

Using the Surveillance, Epidemiology, and End Results (SEER) database, we conducted univariate and multivariate Cox regression analyses to identify independent prognostic factors for T1–T2 SGSCC. We also enrolled 109 patients with T1–T2 SGSCC from the First Affiliated Hospital of Xinjiang Medical University as an external validation set. In addition, we developed a nomogram to predict the prognosis of T1–T2 SGSCC, assessed the predictive accuracy and discriminatory ability of the nomogram using the area under the curve (AUC), C-index, receiver operating characteristic (ROC) curve and calibration curve, and confirmed the clinical validity of the nomogram using decision curve analysis (DCA).

**Results:**

Our investigation identified nine prognostic indicators for T1–T2 SGSCC: age (≥ 65 years), marital status, American Joint Committee on Cancer (AJCC) stage (II–IV), grade (III–IV), M stage (M1), radiotherapy, chemotherapy, sex (female), and surgery. These variables were used to create accurate nomograms that predict overall and specific survival rates at 1, 3, and 5 years. The nomograms demonstrated superior prognostic value and accuracy compared to AJCC staging. Laryngectomy with partial laryngectomy is the preferred treatment option for T1–T2 SGSCC cases, providing superior overall survival (OS) and cancer-specific survival (CSS). Radiotherapy also improves OS and CSS. Our results were based on a comprehensive analysis of various indicators, including the C-index, ROC curve, calibration curve, and DCA curve.

**Conclusion:**

Nomograms provide significant advantages in treatment decision making and diagnosis. Laryngectomy with partial laryngectomy is the most appropriate method for T1–T2 SGSCC cases. However, radiotherapy can also be used. Thus, patients with T1–T2 SGSCC should be evaluated to determine if combination therapy is the optimal treatment approach. Nevertheless, further research is needed to understand the role of chemotherapy. Overall, this study identified nine key predictors of future outcomes, aiding healthcare professionals in assessing risks and making treatment decisions for T1–T2 SGSCC patients.

## Introduction

The incidence of laryngeal cancer squamous cells (LSCC) and the rate of fatalities are both increasing, making it one of the most common malignant tumors affecting the head and neck region [1, 2]. LSCC has an annual rate of about 1,700,000 cases worldwide, resulting in a significant number of deaths [3]. LSCC is classified into various forms based on its anatomy, such as supraglottic squamous carcinoma (SGSCC), glottic squamous cell carcinoma, subglottic squamous cell carcinoma, and others [4].

The main factors contributing to the poor prognosis of LSCC include localized invasion, metastasis to cervical lymph nodes, and treatment resistance [5, 6]. A research study conducted in the United States on over 160,000 patients diagnosed with LSCC found that the majority of cases (60–70%) originated from the glottis, while around 35% originated from the supraglottis [4, 7]. SGSCC, in its initial stages, is characterized by tumors classified as stage T1, which are restricted to a specific region of the supraglottis and do not affect vocal cord function. Tumors that spread across multiple areas of the supraglottis or extend beyond the supraglottis or glottis without laryngeal fixation are considered stage T2 SGSCC [8, 9]. Notably, SGSCC is poorly differentiated, exhibits rapid growth, and does not present obvious initial signs [10]. Therefore, early recognition and prompt intervention can increase the survival rates of patients.

During treatment, the goal is to protect the patient’s laryngeal vocalizations and other body processes [11]. However, surgical resection, postoperative radiotherapy, and chemotherapy, which are the most common treatment options for early-stage SGSCC, are not 100% effective in preventing disease progression, recurrence, or death. Notably, recent studies have shown that LSCC is more common in men than in women [12]. Ethnic differences have also been identified [13]. Additionally, various factors, including age, stage, grade, marital status, chemotherapy, radiotherapy, N stage, and surgical procedure, can significantly affect patients’ likelihood of survival. Hence, accurate estimation of the prognosis of SGSCC patients is extremely helpful in developing appropriate treatment programs and follow-up care.

The TNM staging method, developed by the AJCC, is widely used to assess the prognosis of different types of cancer and to guide treatment decisions. Nevertheless, TNM staging only considers a limited number of factors, excluding specific clinical factors that may impact the outcome. Moreover, limited research has been conducted to determine the connection between prognosis models and T1–T2 SGSCC. Therefore, it is crucial for doctors to use a predictive assessment method that takes into account all relevant elements, allowing for appropriate treatments and effective communication with patients and families.

Nomograms that include several independent factors to predict survival are usually more accurate and user-friendly in clinical settings [14]. In this study, we used data from the SEER database and verified cases of stage T1–T2 SGSCC from the First Affiliated Hospital of Xinjiang Medical University to develop a reliable nomogram. Robust statistical algorithms based on standard clinicopathological guidelines were used in this process. The nomogram was created to predict the outcome of patients with SGSCC in the T1–T2 phase by identifying factors that predict OS and CSS through thorough statistical analysis. The effectiveness of the nomograms was then assessed.

## Materials and methods

The study received ethical approval from the Ethics Committee of First Affiliated Hospital of Xinjiang Medical University. Patient information used in the study was anonymized to protect their privacy. No ethical approval was required for the use of information in the SEER Program.

### Patients

This study included individuals with T1–T2 SGSCC who sought medical attention at the First Affiliated Hospital of Xinjiang Medical University between 2010 and 2021 as well as were recorded in the SEER database between 2010 and 2018.

#### Inclusion criteria

Participants were selected based on their diagnoses recorded in the SEER database between 2010 and 2018 and screened at the First Affiliated Hospital of Xinjiang Medical University between 2010 and 2021. The primary site of the neoplasm was confirmed to be the supraglottic zone (C32.1-Supraglottis). Participants were identified as having squamous-cell cancer based on the International Disease Oncology Code (ICD-O-3) guidelines, which were confirmed by pathological examination. The identified types of squamous-cell cancer included 8070/3 squamous cell carcinoma, NOS; 8071/3 squamous cell carcinoma, keratinized squamous cancer, large cells, non-keratinizing; and 8073/3 squamous cell cancer, small-cell, non-keratinizing.

#### Exclusion criteria

1. Duplicate or incomplete patient profiles, such as cases with unidentified marital status or race details.
2. Patients diagnosed with non-T1–T2 SGSCC.
3. Patients with limited follow-up durations and insufficient survival data.
4. Patients with treatment plans lacking comprehensive details, including specifics on the procedure employed to treat the unknown primary site, as well as details on radiotherapy and chemotherapy.

**Fig. 1:**
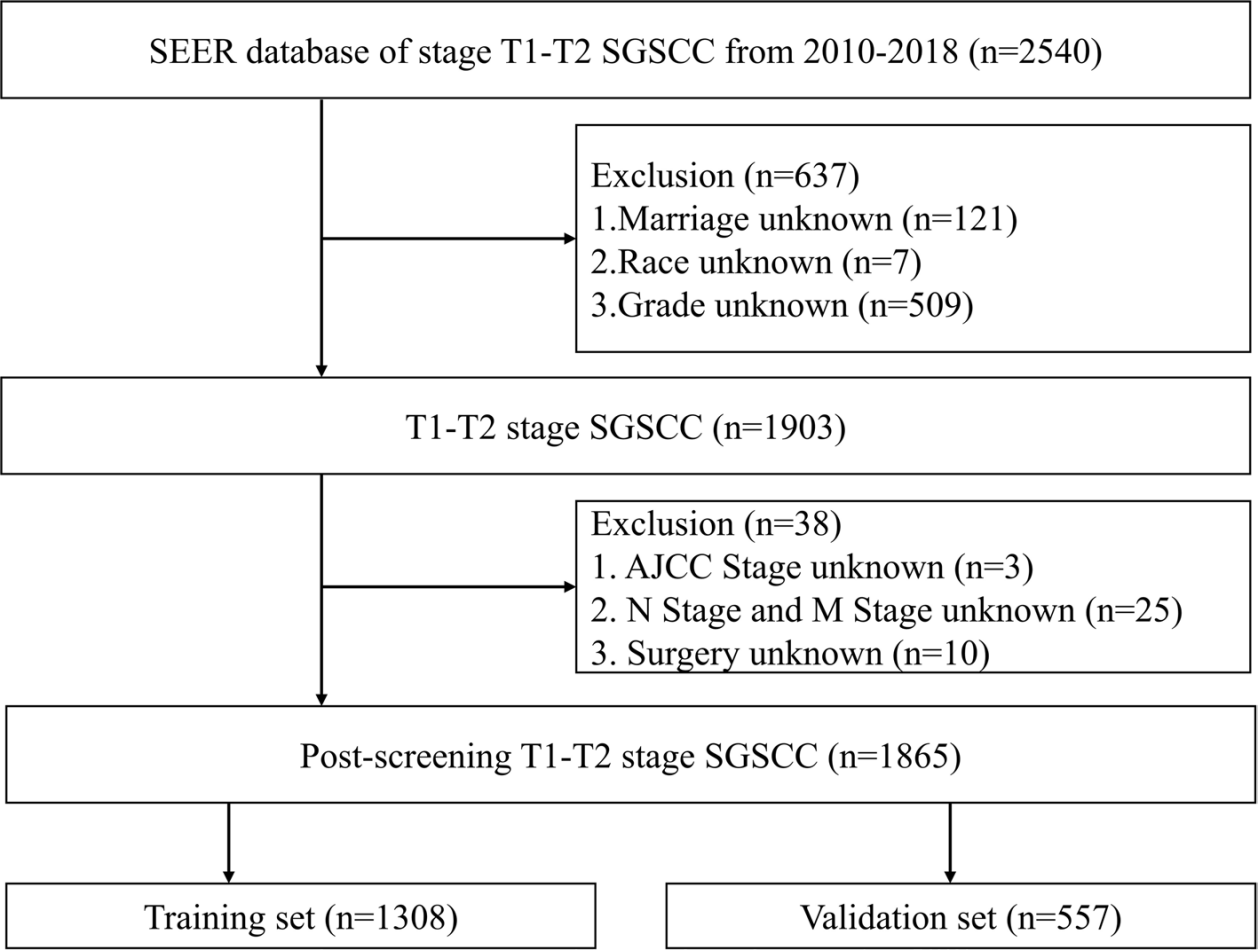
Flow chart developed to screen cases of T1–T2 supraglottic squamous cell carcinoma using data acquired from the SEER database

**Fig. 2:**
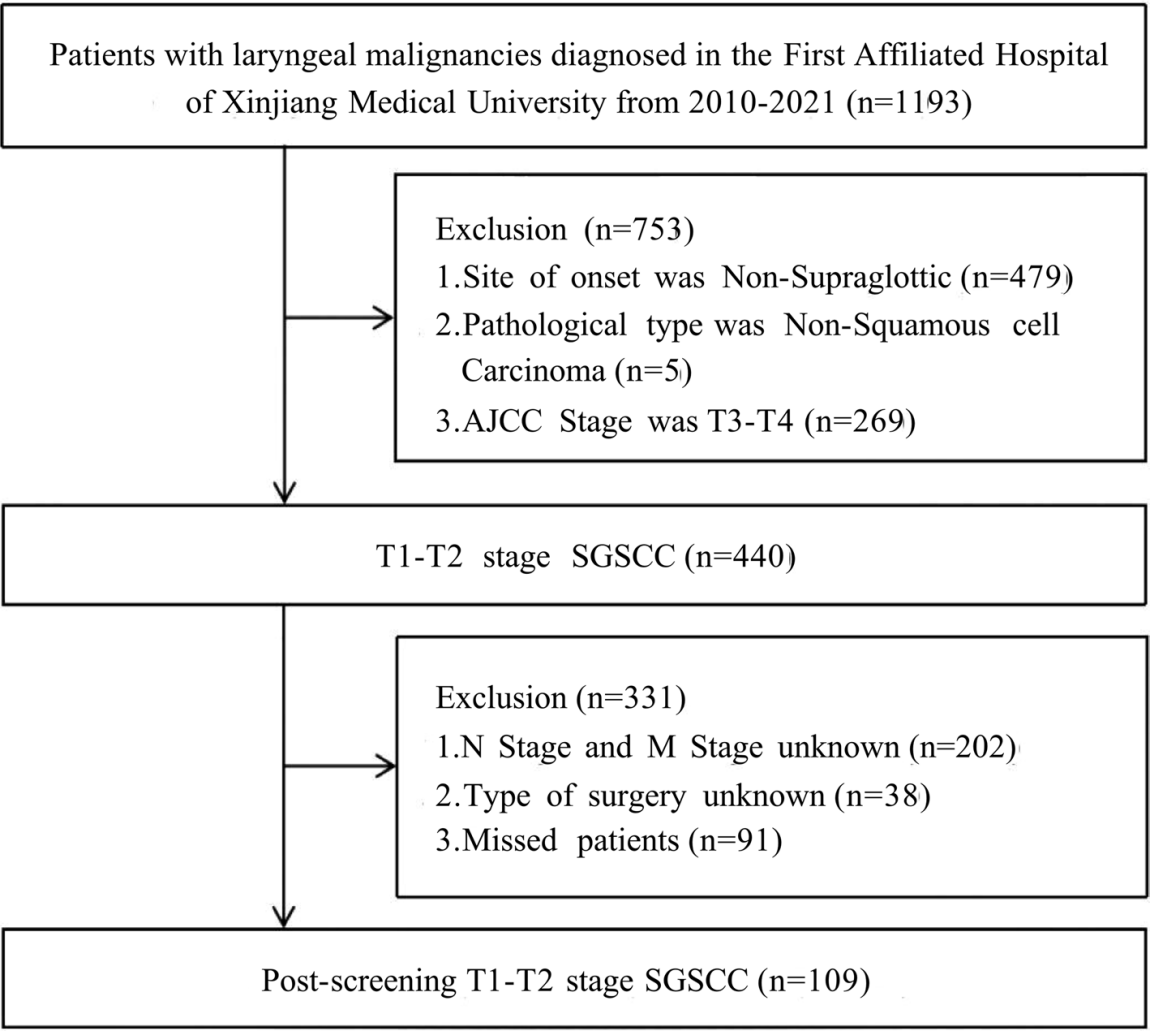
Flow chart developed to screen cases of confirmed T1–T2 stage supraglottic squamous cell carcinoma using data acquired from the First Affiliated Hospital of Xinjiang Medical University from 2010 to 2021

This study involved examining patients in both the SEER database and the database from the First Affiliated Hospital of Xinjiang Medical University using established standards for exclusion and inclusion. The selection process included a cohort of 1865 patients and 109 qualified patients identified with T1–T2 SGSCC. Strict screening procedures were used to determine the proportionality of our results to the population being studied. The patient selection process is illustrated in the flow charts shown in Figs. 1 and 2.

### Data collection

This study investigated various aspects, including the patient’s age and tumor stage (according to the AJCC staging), sex, tumor grade, marital status, race, summary stage, M stage, N stage, primary cancer surgery, radiation, chemotherapy, and survival rate. The 8th edition of the AJCC staging method was applied to classify all clinical factors.

The surgical techniques for addressing primary tumors were categorized into several groups, including non-operative methods, local intervention, partial laryngectomy, total laryngectomy, and other forms of laryngectomy.

- Local intervention group:
  1. Local tumor excision, NOS. Code: 20
  2. Laser ablation. Code: 24 + laser excision and 25
  3. Excisional biopsy. Code: 27
- Partial laryngectomy group:
  1. Partial excision of the primary site, NOS; subtotal/partial laryngectomy, NOS; hemilaryngectomy, NOS. Code: 30
  2. Vertical laryngectomy. Code: 31
  3. Supraglottic laryngectomy. Code: 33
- Total laryngectomy group:
  1. Total or radical laryngectomy, NOS. Code: 40
  2. Total laryngectomy ONLY. Code: 41
  3. Radical laryngectomy ONLY. Code: 42
- Other types of laryngectomy:
  1. Pharyngolaryngectomy. Code: 50
  2. Laryngectomy, NOS. Code: 80
  3. Surgery, NOS. Code: 90

### Statistical analysis

The study used the Kaplan–Meier method to perform an analysis of survival. A total of 1865 patients were randomly divided into the training set and the validation set 1 with a proportion of 7 to 3. The aim was to ensure the accuracy and reliability of the prognostic assessment model and the experimental results. Additionally, external validation was conducted using the data of 109 patients from the First Affiliated Hospital of Xinjiang Medical University.

Multivariate and univariate Cox regression analyses were performed to identify the specific risks associated with the OS of patients with T1–T2 SGSCC. The nomograms were designed and visualized using R Studio, and the “rms” function was used to evaluate the prognostic value in the nomograms. C-index values, calibration curves, and ROC curves were calculated and constructed. The AUC values derived from the ROC curves ranged from 0.5 to 1, with higher values indicating superior discriminatory capabilities. The bootstrap method was utilized for both internal and external tests on the validation and training sets.

The predictive abilities of the nomogram plot were thoroughly examined using the C-index, calibration curve, and DCA. The C-index is a metric that measures the precision of the prediction, with higher numbers indicating better precision. The calibration curve and principal component analysis provided additional insight into the prognostic capabilities of the nomogram chart. These techniques were applied to verify the accuracy and relevance of the findings. The most optimal value for the C-index is 1, indicating 100% accuracy in prediction, while the lowest value is 0.5. The calibration curve measures the agreement between observed and predicted incidence, with a higher degree of convergence indicating a more precise nomogram. A standard threshold of P < 0.05 (two-tailed) was used to establish the statistical significance of the tests.

## Results

### Basic patient characteristics

Patients (n = 1865) were chosen from the SEER database based on inclusion and exclusion criteria. The “caret” R software package was used to randomly assign the patients into two clusters. The first cluster consisted of a training set (n = 1308, 70.13%). The second cluster was a validation set 1 (n = 557, 29.87%). To further confirm the results, we selected patients who met the inclusion criteria from the available data at the First Affiliated Hospital of Xinjiang Medical University (validation set 2, n = 109). Tables 1 and 2 provide details on the general characteristics and the characteristics of the three patient categories, respectively.

**Table 1:** Demographic, clinical characteristics of patients with stage T1–T2 SGSCC in the training and validation set 1 in SEER

Variables	Level	Overall	Training set	Validation set 1
n		1865	1308	557
Age (%)	< 65	1111 (59.6)	779 (59.6)	332 (59.6)
	≥ 65	754 (40.4)	529 (40.4)	225 (40.4)
Sex (%)	Male	1217 (65.3)	851 (65.1)	366 (65.7)
	Female	648 (34.7)	457 (34.9)	191 (34.3)
Marital (%)	Married	841 (45.1)	590 (45.1)	251 (45.1)
	Unmarried	1024 (54.9)	718 (54.9)	306 (54.9)
Race (%)	Caucasian	1541 (82.6)	1077 (82.3)	464 (83.3)
	African American	267 (14.3)	194 (14.8)	73 (13.1)
	Other racial groups	57 (3.1)	37 (2.8)	20 (3.6)
Grade (%)	I	161 (8.6)	107 (8.2)	54 (9.7)
	II	1118 (59.9)	789 (60.3)	329 (59.1)
	III	576 (30.9)	403 (30.8)	173 (31.1)
	IV	10 (0.5)	9 (0.7)	1 (0.2)
Summary stage (%)	Localized	804 (43.1)	557 (42.6)	247 (44.3)
	Regional	883 (47.3)	625 (47.8)	258 (46.3)
	Distant	178 (9.5)	126 (9.6)	52 (9.3)
AJCC stage (%)	I	392 (21.0)	271 (20.7)	121 (21.7)
	II	559 (30.0)	388 (29.7)	171 (30.7)
	III	286 (15.3)	199 (15.2)	87 (15.6)
	IV	628 (33.7)	450 (34.4)	178 (32.0)
N (%)	N0	964 (51.7)	668 (51.1)	296 (53.1)
	N1	302 (16.2)	211 (16.1)	91 (16.3)
	N2	550 (29.5)	398 (30.4)	152 (27.3)
	N3	49 (2.6)	31 (2.4)	18 (3.2)
M (%)	M0	1784 (95.7)	1251 (95.6)	533 (95.7)
	M1	81 (4.3)	57 (4.4)	24 (4.3)
Surg (%)	Non-operated	1441 (77.3)	1011 (77.3)	430 (77.2)
	Local intervention	186 (10.0)	117 (8.9)	69 (12.4)
	Partial laryngectomy	148 (7.9)	116 (8.9)	32 (5.7)
	Total laryngectomy	69 (3.7)	47 (3.6)	22 (3.9)
	Other types of laryngectomy	21 (1.1)	17 (1.3)	4 (0.7)
Radiation (%)	No/unknown	353 (18.9)	248 (19.0)	105 (18.9)
	Yes	1512 (81.1)	1060 (81.0)	452 (81.1)
Chemotherapy (%)	No/unknown	937 (50.2)	662 (50.6)	275 (49.4)
	Yes	928 (49.8)	646 (49.4)	282 (50.6)

**Table 2:** Demographic and clinical characteristics of patients with T1–T2 stage SGSCC in validation set 2 from the data of the First Affiliated Hospital of Xinjiang Medical University

Variables	Level	Overall
n		109
Age (%)	< 65	62 (56.9)
	≥ 65	47 (43.1)
Sex (%)	Male	101 (92.7)
	Female	8 (7.3)
Marital (%)	Married	109 (100.0)
	Unmarried	0 (0)
Race (%)	–	–
Grade (%)	I	75 (68.8)
	II	24 (22.0)
	III	10 (9.17)
	IV	0 (0.0)
AJCC stage (%)	I	30 (27.5)
	II	51 (46.8)
	III	13 (11.9)
	IV	15 (13.8)
N (%)	N0	81 (74.3)
	N1	14 (12.8)
	N2	14 (12.8)
	N3	0 (0)
M (%)	M0	106 (97.2)
	M1	3 (2.8)
Surg (%)	Non-operated	24 (22.0)
	Local intervention	19 (17.4)
	Partial laryngectomy	60 (55.1)
	Total laryngectomy	6 (5.5)
	Other types of laryngectomy	0 (0)
Radiation (%)	No/unknown	38 (34.9)
	Yes	71 (65.1)
Chemotherapy (%)	No/unknown	83 (74.1)
	Yes	26 (23.9)

From the SEER database, 1865 patients were included in the analysis. Of these patients, 1111 (59.6%) were below the age of 65, while 754 (40.4%) were 65 years and older. The study sample comprised 1865 patients, with 1217 (65.3%) men and 648 (34.7%) women. Regarding marital status, 841 were married (45.1%), while 1024 were unmarried (54.9%). The study population included 1865 patients, with 1541 (82.6%) identifying as white, 267 (14.3%) as black and 57 (3.1%) belonging to other racial groups. According to tumor grade classification, 161 cases (8.6%) were grade I, 1118 cases (59.9%) were grade II, 576 cases (30.9%) were grade III, and 10 cases (0.5%) were grade IV. In terms of summary stage, 804 cases (43.1%) were confined to a specific area, 883 cases (47.3%) were regional, and 178 cases (9.5%) were distant. Regarding the AJCC stage, 392 cases (21.0%) were stage I, 559 cases (30.0%) were stage II, 286 cases (15.3%) were stage III, and 628 cases (33.7%) were stage IV. In terms of N stages, 964 cases (51.7%) were N0, 302 cases (16.2%) were N1, 550 cases (29.5%) were N2, and 49 cases (2.6%) were N3. Regarding M stages, 1784 cases (95.7%) were M0, and 81 cases (4.3%) were M1. A total of 1441 patients did not undergo surgery (77.3%), 186 patients received local intervention (10.0%), 148 patients underwent partial laryngectomy (7.9%), 69 patients underwent total laryngectomy (3.7%), and 21 patients underwent other surgical methods (1.1%). In total, 1512 patients underwent radiotherapy treatment (81.1%), while 353 patients did not receive radiotherapy (18.9%). Additionally, 928 patients were treated with chemotherapy (49.8%), while 937 were not (50.2%).

In the cohort of patients collected from the First Affiliated Hospital of Xinjiang Medical University, the majority (n = 62, 56.9%) were under 65 years old, while 47 patients (43.1%) were over 65 years old. The sample size consisted of 109 patients, with a predominance of men at 101 patients (92.7%) and a minority of women at 8 patients (7.3%). Furthermore, all 1009 patients in the entire sample were found to be married. According to tumor grade, grade I comprised the largest portion of patients at 68.8% (n = 75), followed by grade II at 22.0% (n = 24), and then grade III at 9.17% (n = 10). The study did not find any instances of grade IV. Regarding the AJCC stage, stage I comprised 27.5% (n = 30) of cases, stage II comprised 46.8% (n = 51) of cases, stage III comprised 11.9% (n = 13) of cases, and stage IV comprised 13.8% (n = 15) of cases. In terms of different stages, N0 had the highest percentage at 74.3% (n = 81), while N1 and N2 accounted for 12.8% each, with 14 cases for each stage. No instances of N3 were detected. M0 was the most common in terms of M stages, accounting for 97.2% of the sample with 106 patients. On the other hand, M1 constituted a negligible fraction, making up only 2.8% of the total instances. The sample consisted of 109 patients, 24 of whom had not undergone surgery, making up 22.0% of the population. There were 19 patients who underwent local interventions, accounting for 17.4% of the total. The majority of patients (55.1%) underwent partial laryngectomy (n = 60), while a lower proportion underwent partial laryngectomy. Only 5.5% required a total laryngectomy (n = 6). No other procedures were used in the cohort. Out of the total number of patients, 71 received radiotherapy, constituting 65.1% of the patients. On the other hand, 38 patients did not receive radiotherapy, accounting for 34.9% of the population. Chemotherapy was offered to 26 patients, representing 23.9% of the patients. The remaining 83 patients (74.1%) did not receive chemotherapy.

To ensure the stability of the experimental data, the Chi-square test was used to analyze the basic data characteristics of the validation sets and training set. The above variables were found to be independent and have no relationship with each other, as shown in Tables 1 and 2.

### Univariate analysis

Univariate Cox regression analysis was utilized to identify the general variables of patients in the training set, separating them into OS group and CSS group. The aim was to identify prognostic factors affecting patients with T1–T2 SGSCC.

In the OS group several factors were significantly associated with T1–T2 SGSCC prognosis, including age (age ≥ 65), sex (female), marital status (unmarried), AJCC stage III and IV, grade IV, and other variables such as AJCC stage III combined with grade IV, T1, M1, N1, N2, N3, radiotherapy, partial laryngectomy, and distal and regional summary stages (P < 0.05). However, variables such as AJCC stage II, chemotherapy, grade II, race, local intervention, other types of laryngectomy, and total laryngectomy did not have an impact on patient outcomes (P > 0.05) (Table 3).

**Table 3:** Univariate analysis of the OS and CSS groups in the training set

Variables	OS		CSS	
	HR.CI95	*P*	HR.CI95	*P*
Age				
< 65	Ref		Ref	
≥ 65	1.86 (1.58–2.19)	< 0.001	1.63 (1.33–1.99)	< 0.001
AJCC stage				
I	Ref		Ref	
II	1.18 (0.91–1.52)	0.208	1.9 (1.29–2.79)	0.001
III	1.4 (1.05–1.87)	0.022	2.69 (1.78–4.05)	< 0.001
IV	1.93 (1.52–2.46)	< 0.001	4.05 (2.82–5.81)	< 0.001
Chemotherapy				
No	Ref		Ref	
Yes	1.05 (0.89–1.24)	0.557	1.27 (1.04–1.55)	0.02
Grade				
I	Ref		Ref	
II	1.13 (0.83–1.54)	0.429	1.66 (1.05–2.63)	0.029
III	1.27 (0.92–1.76)	0.141	2.19 (1.37–3.5)	0.001
IV	2.39 (1.13–5.08)	0.023	4.43 (1.87–10.49)	0.001
Martial				
Married	Ref		Ref	
Unmarried	1.67 (1.41–1.98)	< 0.001	1.78 (1.45–2.2)	< 0.001
AJCC T stage				
T1	Ref		Ref	
T2	1.24 (1.04–1.48)	0.017	1.52 (1.21–1.91)	< 0.001
AJCC N stage				
N1	1.37 (1.09–1.72)	0.007	1.88 (1.42–2.48)	< 0.001
N2	1.55 (1.29–1.87)	< 0.001	2.22 (1.76–2.79)	< 0.001
N3	2.6 (1.59–4.26)	< 0.001	4.52 (2.69–7.6)	< 0.001
AJCC M stage				
M0	Ref		Ref	
M1	6.29 (4.66–8.49)	< 0.001	8.7 (6.31–11.98)	< 0.001
Race				
White	Ref		Ref	
Black	1.18 (0.95–1.47)	0.14	1.31 (1.01–1.71)	0.043
Other	0.97 (0.59–1.59)	0.891	1.33 (0.78–2.27)	0.301
Radiation				
No	Ref		Ref	
Yes	0.63 (0.52–0.76)	< 0.001	0.7 (0.55–0.89)	0.003
Sex				
Male	Ref		Ref	
Female	0.83 (0.7–0.99)	0.034	0.78 (0.63–0.96)	0.021
Summary stage				
Localized	Ref		Ref	
Distant	3.28 (2.57–4.19)	< 0.001	5.34 (4.01–7.1)	< 0.001
Regional	1.26 (1.05–1.5)	0.013	1.7 (1.35–2.15)	< 0.001
Surgery				
Non-operated	Ref		Ref	
Partial laryngectomy	0.45 (0.32–0.64)	< 0.001	0.31 (0.18–0.52)	< 0.001
Local intervention	0.79 (0.59–1.06)	0.116	0.75 (0.52–1.08)	0.119
Other types of laryngectomy	1.45 (0.83–2.51)	0.19	1.81 (0.99–3.3)	0.054
Total laryngectomy	0.72 (0.46–1.14)	0.163	0.79 (0.46–1.35)	0.394

The primary factors for predicting the CSS group were sex, age, and marital status. Other factors considered included tumor grade (AJCC stage II–IV) and tumor stage (II–IV), M1 stage, N1–N3 stage, distal and regional summary stages, as well as chemotherapy, radiotherapy, and partial laryngectomy. These variables showed a statistically significant correlation with CSS (P < 0.05). Race, local intervention, other types of laryngectomy, and total laryngectomy had no effect on patient outcomes (P > 0.05) (Table 3).

### Prognostic survival analysis

#### Survival analysis of prognostic factors in the OS group

**Fig. 3:**
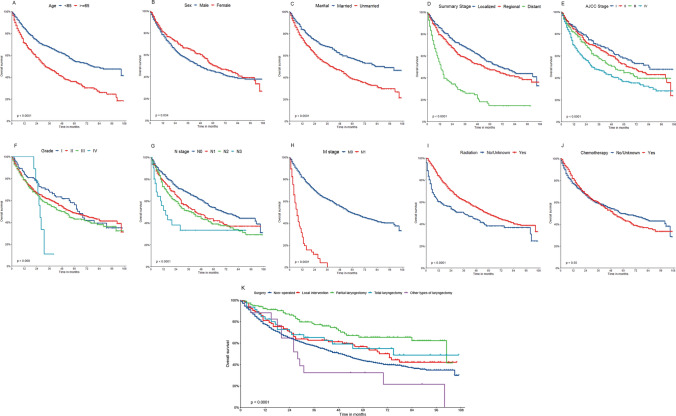
KM curve analyses for patients in the OS group, examining the influence of various factors on prognosis: **A** age (P < 0.001). **B** Sex (P = 0.034). **C** Marital status (P < 0.001). **D** Summary stage (P < 0.001). **E** Tumor stage (P < 0.001). **F** Grade (P < 0.001). **G** N stage (P < 0.001). **H** M stage (P < 0.001). **I** Radiotherapy (P < 0.001). **J** Chemotherapy prescriptions (P = 0.55). **K** Surgical procedures (P < 0.001)

The survival curve (KM curve) of patients in the OS group was drawn using R Studio and the KM method. The results are shown in Fig. 3. The results indicated that age, sex, marital status, summary stage, tumor stage, tumor grade, N stage, M stage, radiotherapy, and different surgical methods were significant factors (P < 0.05).

#### Survival analysis of prognostic factors in patients with CSS

**Fig. 4:**
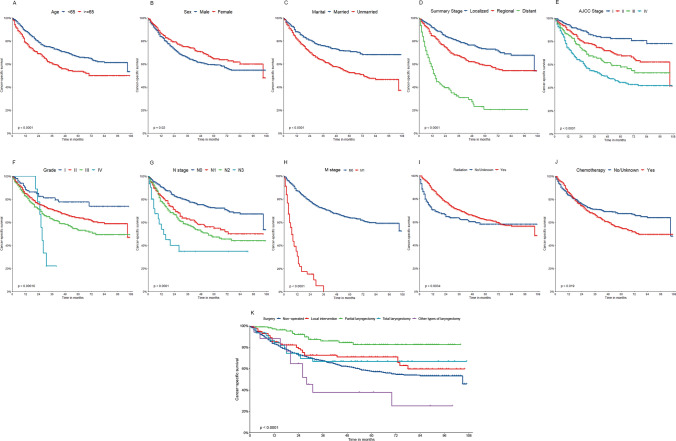
KM curve analyses for patients in the CSS group, examining the influence of various factors on prognosis: **A** age (P < 0.001). **B** Sex (P = 0.02). **C** Marital status (P = 0.001). **D** Summary stage (P < 0.001). **E** Tumor stage (P < 0.001). **F** Grade (P = 0.00016). **G** N stage (P < 0.001). **H** M stage (P < 0.001). **I** Radiotherapy (P = 0.0034). **J** Chemotherapy (P = 0.019). **K** Surgical techniques (P < 0.001)

This research evaluated the significance of different factors in the CSS group with respect to the survival times of patients. The KM technique was used to generate and display the KM curves of patients from the validation set 1 (Fig. 4).

#### Survival analysis of prognostic factors of patients in validation set 2 from the First Affiliated Hospital of Xinjiang Medical University

**Fig. 5:**
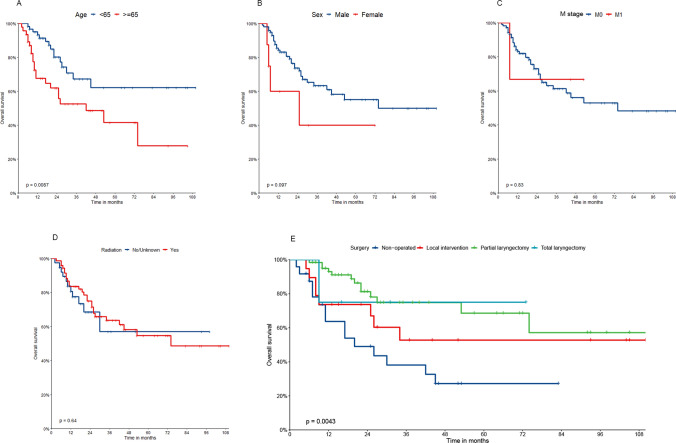
KM curve analyses for patients in validation set 2, examining the influence of various factors on prognosis: **A** age (P < 0.001). **B** Sex (P = 0.097). **C** M stage (P = 0.83). **D** Radiotherapy (P = 0.064). **E** Surgical procedures (P = 0.0043)

The same procedure was used to evaluate the significance of each factor in validation set 2 in terms of patients' survival time. The KM method was employed to generate and extend the KM curve for the patients in the validation set 2 (Fig. 5).

### Multivariate analysis

**Fig. 6:**
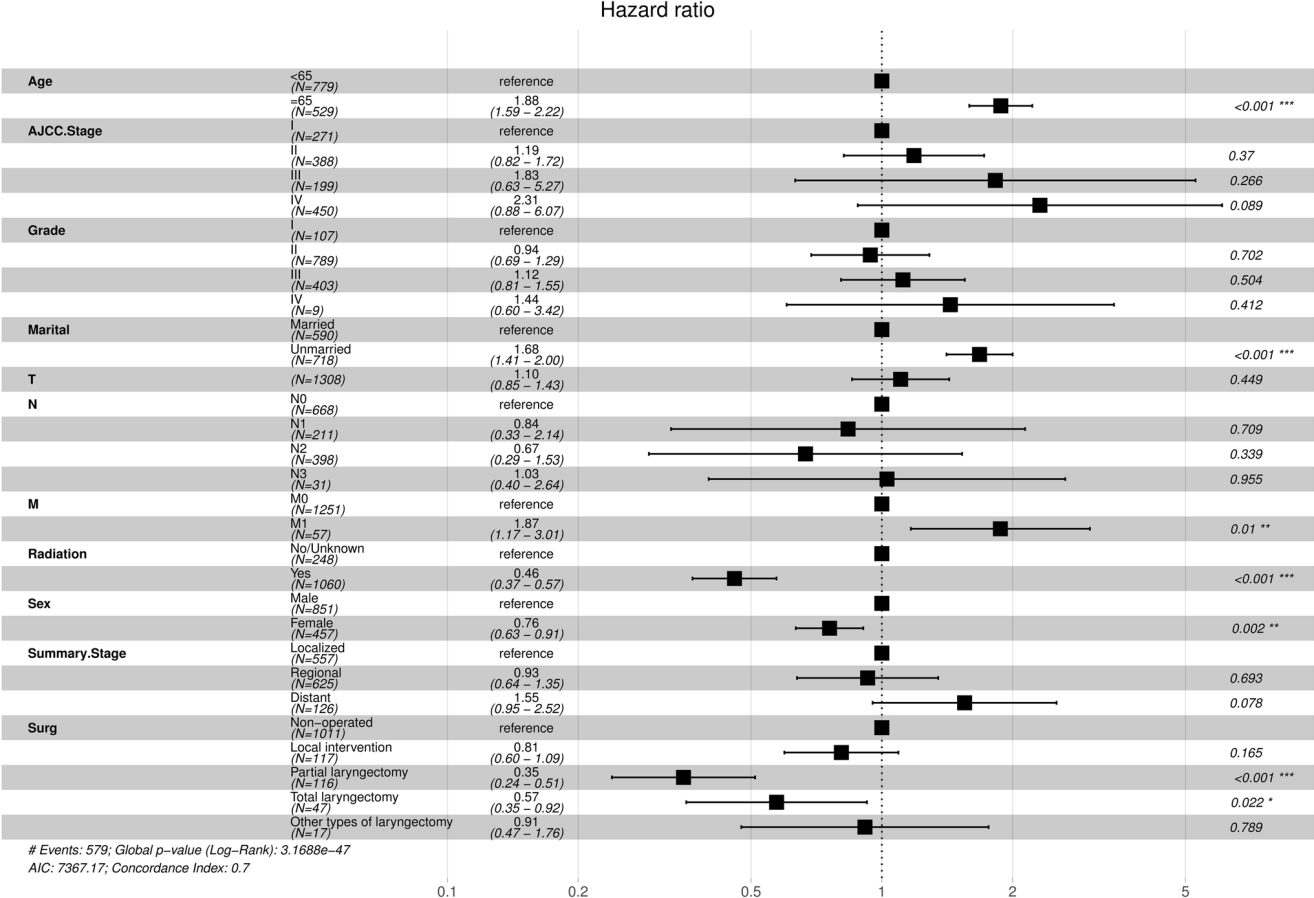
Multivariate Cox regression forest plot for the OS group

Multivariate Cox regression analysis was performed to validate the results. In the OS group, the study found that age (age ≥ 65), sex, marital status (unmarried), M1 stage, radiotherapy, local intervention, partial laryngectomy, and total laryngectomy were significant prognostic factors (P < 0.05). These factors could be used as independent prognostic risk factors for T1–T2 supraglottic squamous cell carcinoma (Table 4 and Fig. 6).

**Table 4:** Multivariate analysis of the OS and CSS groups in the training set

Variables	OS		CSS	
	HR.CI95	*P*	HR.CI95	*P*
Age				
< 65	Ref		Ref	
≥ 65	1.88 (1.59–2.22)	< 0.001	1.61 (1.3–1.98)	< 0.001
AJCC stage				
I	Ref		Ref	
II	1.19 (0.82–1.72)	0.3696	2.03 (1.24–3.32)	0.005
III	1.83 (0.63–5.27)	0.2664	5.18 (1.57–17.05)	0.0068
IV	2.31 (0.88–6.07)	0.089	5.97 (2.03–17.55)	0.0012
Chemotherapy				
No	Ref		Ref	
Yes	–	–	0.75 (0.58–0.97)	0.0288
Grade				
I	Ref		Ref	
II	0.94 (0.69–1.29)	0.7024	1.26 (0.79–2.01)	0.327
III	1.12 (0.81–1.55)	0.5041	1.76 (1.09–2.83)	0.0198
IV	1.44 (0.6–3.42)	0.4121	1.79 (0.64–5.03)	0.2686
Martial				
Married	Ref		Ref	
Unmarried	1.68 (1.41–2)	< 0.001	1.71 (1.37–2.13)	< 0.001
AJCC T stage				
T1	Ref		Ref	
T2	1.1 (0.85–1.43)	0.4487	1.07 (0.8–1.43)	0.6312
AJCC N stage				
N1	0.84 (0.33–2.14)	0.7086	0.72 (0.27–1.91)	0.5025
N2	0.67 (0.29–1.53)	0.3393	0.64 (0.27–1.48)	0.2958
N3	1.03 (0.4–2.64)	0.9549	1.08 (0.41–2.83)	0.8794
AJCC M stage				
M0	Ref		Ref	
M1	1.87 (1.17–3.01)	0.0095	2.36 (1.42–3.94)	0.001
Race				
White	Ref		Ref	
Black	–	–	1.06 (0.81–1.39)	0.6697
Other	–	–	1.11 (0.64–1.93)	0.7164
Radiation				
No	Ref		Ref	
Yes	0.46 (0.37–0.57)	< 0.001	0.59 (0.44–0.79)	< 0.001
Sex				
Male	Ref		Ref	
Female	0.76 (0.63–0.91)	0.0024	0.73 (0.58–0.91)	0.0053
Summary stage				
Localized	Ref		Ref	
Distant	1.55 (0.95–2.52)	0.0778	1.49 (0.8–2.76)	0.2067
Regional	0.93 (0.64–1.35)	0.6931	0.82 (0.49–1.36)	0.432
Surgery				
Non-operated	Ref		Ref	
Partial laryngectomy	0.35 (0.24–0.51)	< 0.001	0.26 (0.15–0.45)	< 0.001
Local intervention	0.81 (0.6–1.09)	0.1646	0.9 (0.61–1.31)	0.5789
Other types of laryngectomy	0.57 (0.35–0.92)	0.0224	1.32 (0.62–2.82)	0.4784
Total laryngectomy	0.61 (0.38–0.98)	0.04	0.64 (0.37–1.12)	0.1185

**Fig. 7:**
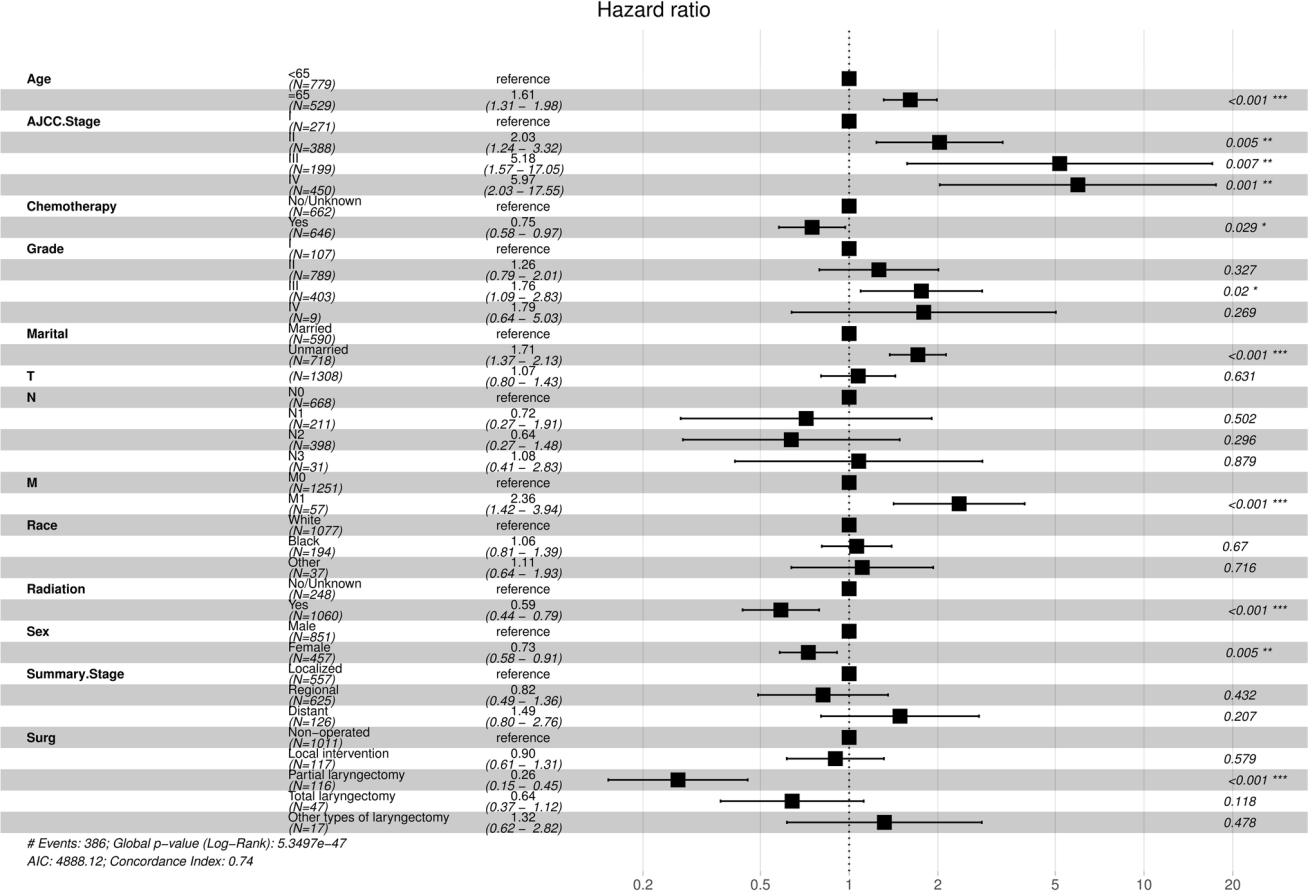
Multivariate Cox regression forest plot for the CSS group

In the CSS group, age (age ≥ 65), sex (female), marital status (unmarried), AJCC stage (stage II–IV), grade III, M1, chemotherapy, radiotherapy, and partial laryngectomy were considered independent predictors (Table 4 and Fig. 7). Multivariate Cox regression analysis can improve the stability of the predictive model.

### Prognostic model for prediction

**Fig. 8:**
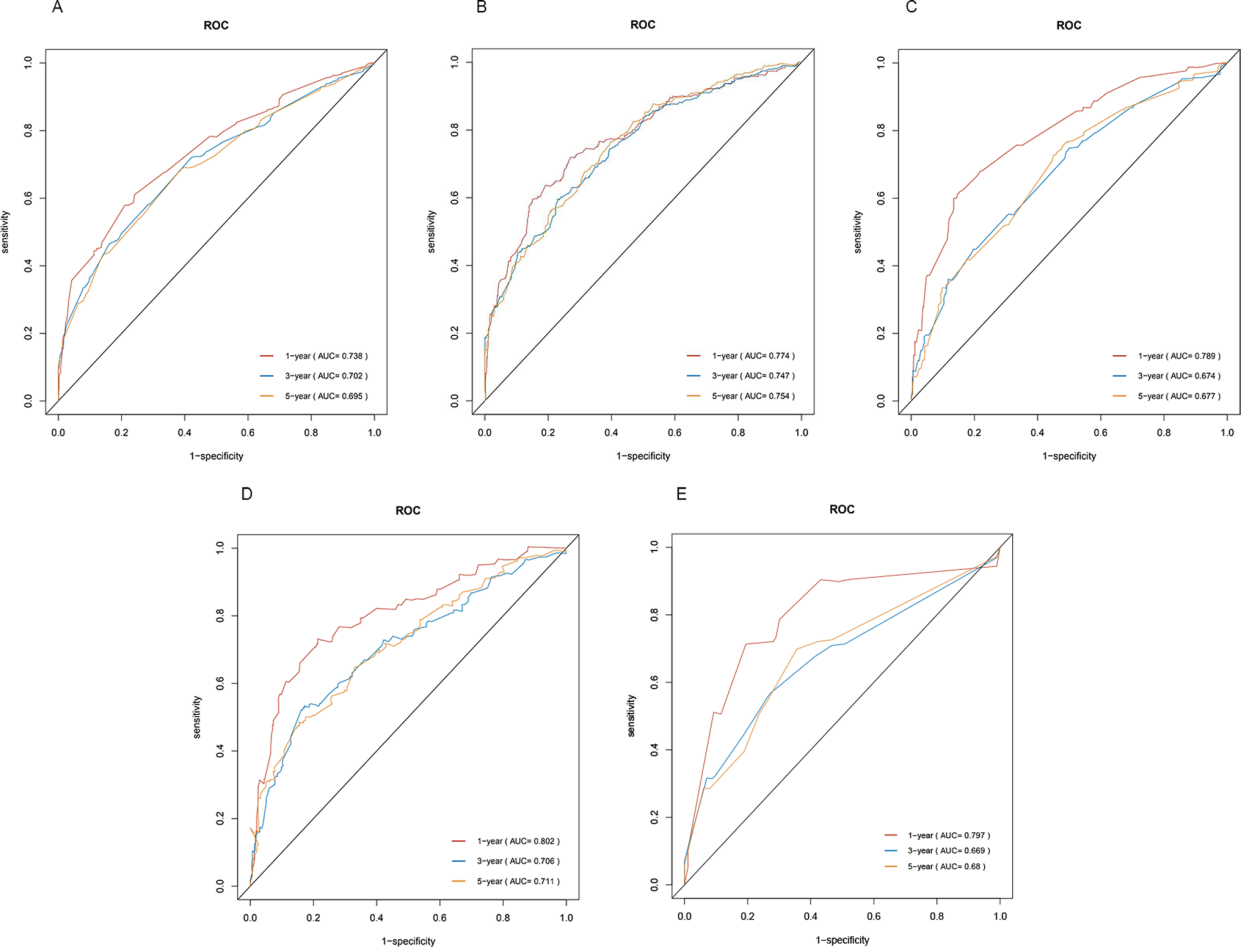
Analysis of patient survival using the multivariate Cox proportional hazards model. The AUC values for the OS and CSS groups at various time points were analyzed utilizing both the training and validation sets and validation set 2. **A** At 1, 3, and 5 years, the OS group in the training set had remarkable AUC values of 0.738, 0.702, and 0.695, respectively. **B** At 1, 3, and 5 years, the AUC values in the CSS group were 0.774, 0.747, and 0.754, respectively. **C** The validation set 1 analysis revealed significantly enhanced performance in the OS group, with remarkable AUC values of 0.789, 0.6743, and 0.66. **D** At 1, 3, and 5 years, the AUC values in the CSS group were 0.802, 0.706, and 0.711, respectively. **E** Validation set 2 further demonstrated the model’s extraordinary accuracy, with AUC values at 1, 3, and 5 years of 0.797, 0.669, and 0.66, indicating outstanding performance

A multivariate Cox prognostic model was developed by selecting reliable variables and then analyzed to determine its efficacy as a diagnostic tool in predicting the prognosis of patients using an ROC curve. As shown in Fig. 8, the 1-, 3-, and 5-year AUC values in the training set’s OS group were 0.738, 0.702, and 0.695, respectively. In the training set’s CSS group, 1-, 3-, and 5-year AUC values were 0.774, 0.747, and 0.754, respectively. The AUC values for the three time points in the OS group of the validation set 1 were 0.789, 0.674, and 0.677, respectively, while those of the CSS group were 0.802, 0.706, and 0.711, respectively. Finally, the AUC values for 1, 3, and 5 years in validation set 2 were 0.797, 0.669, and 0.68, respectively. These results indicate that the prediction model has high accuracy in predicting the outcome of patients with T1–T2 SGSCC.

### Preparation of the model for predicting nomograms: External and internal validation

#### Analysis of the nomogram and drawing of the calibration curve in the OS group

**Fig. 9:**
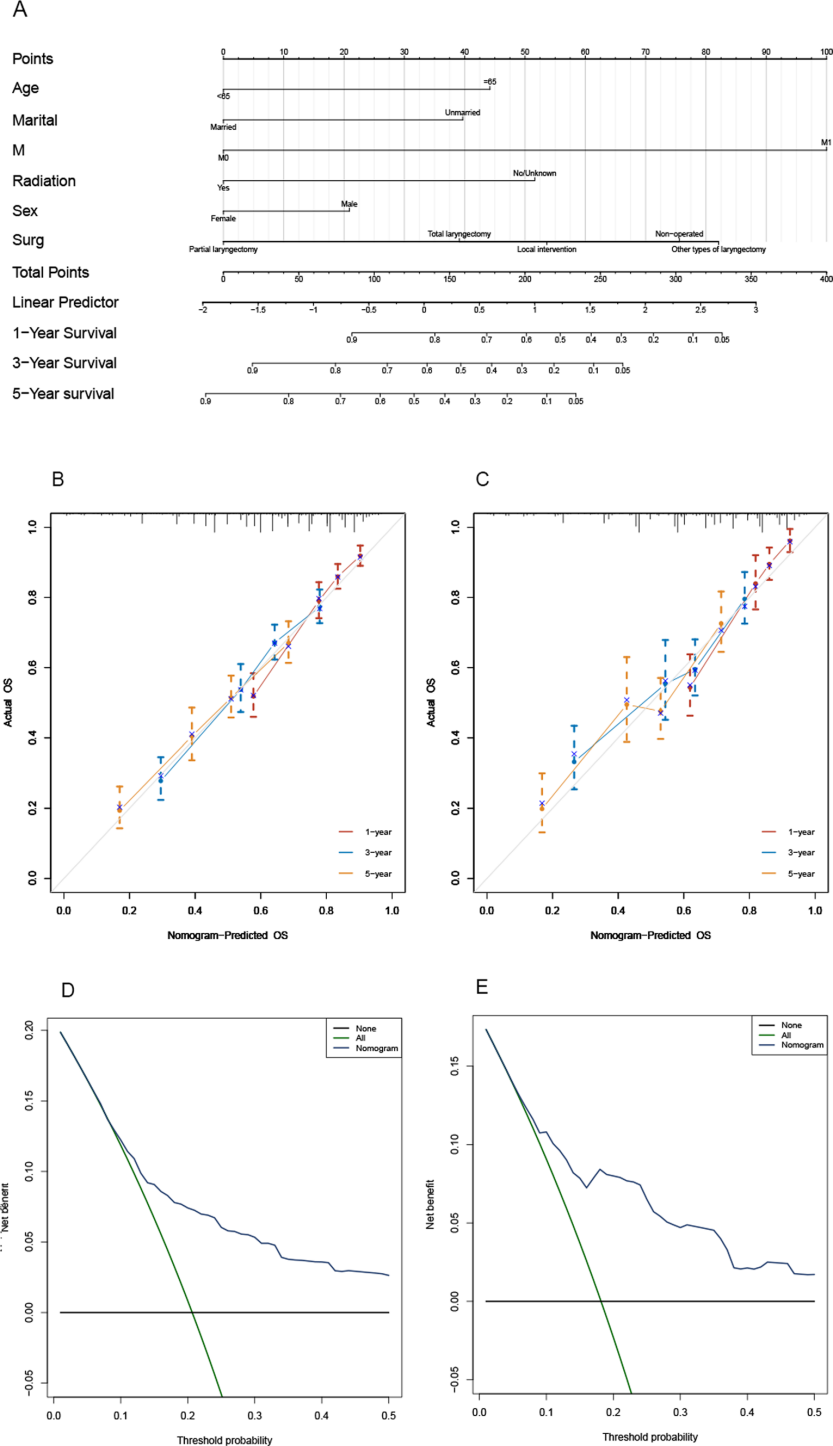
Nomogram plot, calibration curve, and DCA curve of the OS group. **A** Based on the cumulative scores of numerous prognostic risk factors, the 1-year (1Y), 3-year (3Y), and 5-year (5Y) OS rates were determined for patients in both the training and validation sets who belonged to the OS group. **B**, **C** The predictive accuracy of the prognostic model applied to the OS group was evaluated for 1-, 3-, and 5-year OS using calibration curve analysis on the training and validation sets of patients. **D**, **E** DCA curves were plotted for patients in the training and validation sets who belonged to the OS group

Nomogram analysis was used to assign scores to various risk factors, including surgical procedure, age, sex, marital status, stage, and radiation, to provide a more visual representation of the results of this multivariate analysis. The sum of the factor scores was used to determine the total score of the 1-, 3-, and 5-year survival rates for each patient following function transformations, as depicted in Fig. 9A. The C-index scores derived from both external and internal validation for the OS group were obtained from the training set. The internal validation produced an index score of 0.683 (95% CI 0.671–0.695), while the external validation produced an index score of 0.686 (95% CI 0.668–0.705). The calibration curve, shown in Fig. 9B, C, and the DCA curve, depicted in Fig. 9D, E, both exhibit the highest degree of concordance across the validation and training sets. These results demonstrate the model's capacity to accurately predict future events.

#### Nomogram and calibration curve analysis: CSS group

**Fig. 10:**
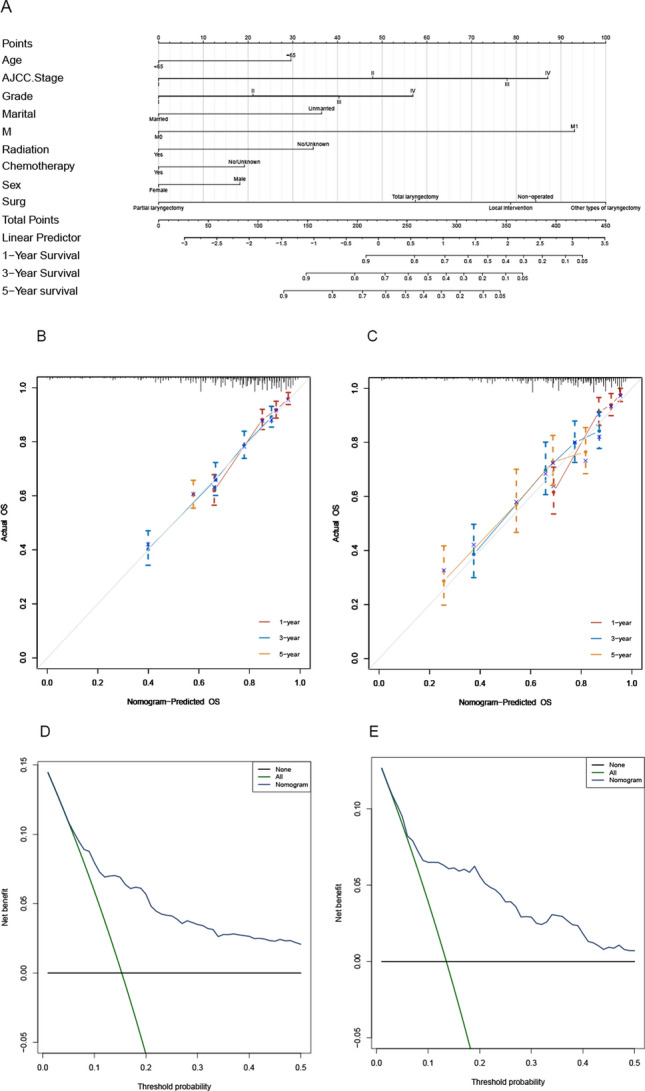
Nomogram plot, calibration curve, and DCA curves for the CSS group. **A** Nomogram analysis was performed to assess the 1-year (1Y), 3-year (3Y), and 5-year (5Y) OS rates among patients belonging to the CSS group in both the training and validation sets, taking into account the cumulative scores of each prognostic risk factor. **B**, **C** In order to evaluate the predictive accuracy of the prognostic model for the 1-year (1Y), 3-year (3Y), and 5-year (5Y) survival rates, calibration curve analysis was performed on the training and validation sets of CSS patients. **D**, **E** DCA curves were plotted for patients in the training and validation sets who belonged to the CSS group

Nomogram analysis was utilized to enhance the clarity of the results obtained from the multivariate analysis. The evaluation considered various risk factors such as surgical method, age, marital status, sex, AJCC grade, degree of chemotherapy treatment, radiation level, and M stage. Each factor was assigned a score, and the survival rates of patients who underwent functional transformation over a period of 3 and 5 years were represented by combining and calculating these scores. Figure 10A displays the outcomes.

For internal validation in the CSS group, the C-index was found to be 0.723 (95% CI 0.709–0.737). External validation with the validation set 1 resulted in a C-index of 0.711 (95% CI 0.689–0.734). Additionally, the calibration curve, represented in Fig. 10B, C, and the DCA curve, illustrated in Fig. 10D, E, both demonstrated an excellent fit in both the validation and training sets. These results suggest that the model has reliable predictive capability.

#### Analysis of the nomogram and drawing of the calibration curve for validation set 2 from the First Affiliated Hospital of Xinjiang Medical University

**Fig. 11:**
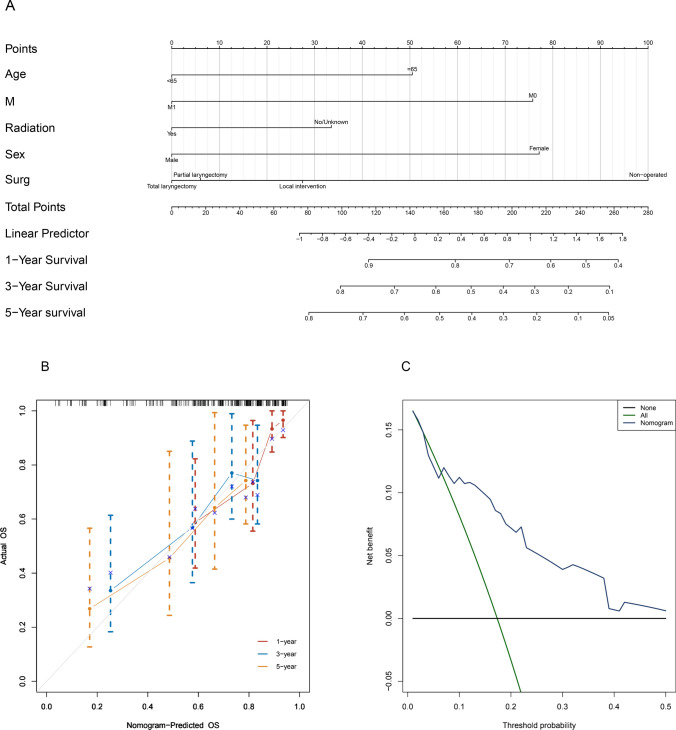
Nomogram plot, calibration curve, and DCA curve for validation set 2. **A** To evaluate the 1-year (1Y), 3-year (3Y), and 5-year (5Y) survival rates among patients in validation set 2, nomogram analysis was performed, taking into account the cumulative scores of each prognostic risk factor. **B** Calibration curve analysis was performed on validation set 2 to assess the predictive accuracy of the prognostic model for the 1-year (1Y), 3-year (3Y), and 5-year (5Y) survival rates. **C** DCA curves were generated for patients in validation set 2

Nomogram analysis was used to assign scores to various risk factors, including surgery method, sex, radiation, and M stage. This was done to enhance the visual presentation of the multivariate analysis results. The total score of the variables was determined and correlated with the 1, 3 and 5-year survival rates for each patient, as shown in Fig. 11A. The calibration and DCA plots of validation set 2 (Fig. 11B, C) showed a good fit to the model, indicating a strong ability to predict the performance of our model.

## Discussion

SGSCC has a poor prognosis because it lacks specific early signs and is often diagnosed at an advanced stage. It also has a high lymph node metastasis rate, resulting from deviating from the clinical stage. Understanding the prognostic factors for SGSCC is crucial for predicting and reducing the rate of this cancer. It can also help in the development and implementation of more effective treatment strategies. However, there is a lack of global research on prognostic indicators for SGSCC. Additionally, many studies have combined information about SGSCC with glottic squamous cells, despite significant differences in their biochemical characteristics, clinical outcomes, and treatments.

According to a prior study, the prognosis of laryngeal cancer is linked to the TNM stage [15]. In addition, according to Mayne et al. [16], consumption of tobacco and alcohol are distinct causes that influence the progression of LSCC. Prompt diagnosis and treatment for this disease can be difficult due to its distinctive pathology and clinical characteristics, leading to the progression to high-grade LSCC and a poor prognosis. Currently, researchers are focusing on analyzing the clinical signs for T1–T2 SGSCC to improve prognosis and provide healthcare professionals with guidance for decision-making and treatment strategies.

The most reliable indicators for prognosis in patients diagnosed with T1–T2 SGSCC include patient age, the resection area, and the tumor stage. However, there is no consensus on possible prognostic factors such as tumor size, chemotherapy, surgery, radiotherapy, and degree of resection. This may be due to the lack of larger sample sizes and multi-institutional studies, as well as differences in medical equipment and expertise among different institutions and professionals.

In this study, extensive analysis was conducted to determine the prognostic factors in patients diagnosed with T1–T2 SGSCC. In contrast to studies that used the SEER database to analyze head and neck diseases and other systemic diseases [17, 18], this study was conducted using both the SEER database and similar cases from the First Affiliated Hospital of Xinjiang Medical University. Subsequently, a nomogram predictive model for prognosis was developed using multivariate and univariate Cox regression analysis. Changes in individuals with early-stage supraglottic cancer were investigated using data from the Denmark Head and Neck Cancer Group [19, 20] and the National Cancer Database. People from various age groups, races, and sexes with different T stages, N stages, and grades were analyzed to determine the likelihood of death from the disease and their OS [20]. Notably, factors such as age, cancer type, T stage, and grade can be utilized to provide insights into patients’ prognoses [21]. Our analysis revealed a significant association between sex and both cancer-specific survival and OS outcomes [22].

Management strategies for early-stage supraglottic cancer include surgical intervention at the primary site and/or radiotherapy, both of which can improve prognosis [23–25]. Primary site surgery has been identified as a cost-effective approach that can be repeated in cases of recurrence. On the other hand, radiotherapy eliminates the need for general anesthesia and has exhibited beneficial functional outcomes [26–28]. However, a Cochrane review revealed no statistically significant differences in survival rates between patients who underwent primary site surgery and those who received radiation therapy for early supraglottic cancer [29]. In this study, both surgery and radiation are being used to treat early-stage supraglottic cancer to evaluate the methods’ effectiveness, as surgery is often successful in removing the cancer and improving outcomes [30].

Recent research has highlighted the potential of radiotherapy as a therapeutic approach for early-stage supraglottic cancer, leading to improved survival rates and local disease control [19, 31, 32]. The efficacy of radiotherapy has been supported by a meta-analysis and systematic review of 1762 patients [33]. Additionally, radiotherapy has been shown to effectively preserve speech outcomes [34]. Our study findings suggest that, given advancements in surgical tools and techniques, partial laryngectomy as a standalone treatment may be the optimal choice for patients with T1–T2 stage SGSCC [26, 35]. Overall, it is recommended to thoroughly evaluate the available treatment options, as both primary site surgery and radiotherapy have shown positive outcomes in managing early-stage supraglottic cancer.

According to research, surgical intervention is considered the most effective approach for relieving localized compression and improving symptoms in individuals with tumors in T1–T2 SGSCC [36]. The extent of tumor resection can vary from total laryngectomy to partial laryngectomy, local intervention, or other types of laryngectomy, depending on the tumor resection area. The postoperative survival period of T1–T2 SGSCC patients is believed to be strongly influenced by the specific surgical technique utilized [37]. However, there is currently no agreement on the precise criteria for surgical resection [36].

Recent research has found that partial laryngectomy is an important prognostic indicator for patients with T1–T2 SGSCC, as evidenced in both multivariate and univariate studies. The KM survival curve developed in this study showed that patients who received partial laryngectomy had a significantly longer survival time compared to those who underwent other surgical treatments or did not undergo any surgical procedures. Some specialists believe that partial laryngectomy may be the best option for T1–T2 SGSCC patients as it can increase survival rates and improve quality of life. Additionally, it promotes functional recovery after surgery and reduces the risk of recurrence [38]. Studies have also shown that the degree of tumor resection is a significant factor in determining the severity of complications in T1–T2 SGSCC patients [39]. In fact, patients who underwent partial surgery experienced better postoperative care compared to those who opted for other interventions [40]. Overall, partial laryngectomy remains the most widely recognized approach in previous studies [41]. However, by utilizing innovative surgical assistance methods, it is possible to develop a surgical plan that protects vital cervical nerves and vessels, thereby reducing the risk of long-term complications.

The most common treatments for patients suffering from stage T1–T2 SGSCC usually include a combination of radiotherapy, chemotherapy, and surgical treatments. However, consensus on the ideal dosage, timing, and efficacy of chemotherapy is lacking, particularly in delivering long-lasting survival benefits [42]. Chemotherapy can be classified into different types based on its specific method of action, such as adjuvant chemotherapy, palliative chemotherapy, and induction chemotherapy. Prior to radiotherapy or surgery, induction chemotherapy is administered to take advantage of the tumor’s extensive vascularization and improve the efficacy of the medication. Adjuvant chemotherapy is commonly used after radiotherapy to target any remaining cancer cells. On the other hand, palliative chemotherapy can be employed for cases of metastatic systemic cancer or treatment recurrence.

The present study confirms the independent prognostic significance of chemotherapy as a risk factor within the CSS group. The results from the KM curve analysis indicate that patients who did not receive chemotherapy had a higher survival rate compared to those who did. Therefore, introducing effective surgical procedures to improve tumor resection, along with timely radiation therapy tailored to each patient's unique medical conditions, could enhance patient survival rates and extend OS durations.

## Conclusions

This study establishes that various factors, including but not limited to age (65 years or older), marital status, AJCC stage (II–IV), tumor grade (II–IV and III–IV), M stage (M1), chemotherapy, radiotherapy, sex (female), and surgical interventions, serve as independent prognostic indicators for patients diagnosed with T1 and T2 SGSCC. Additionally, this study developed an accurate nomogram using data from the SEER database and information from the First Affiliated Hospital of Xinjiang Medical University to determine survival rates at 1 year (1Y), 3 years (3Y), and 5 years (5Y) for patients diagnosed with T1–T2 SGSCC. The nomogram was validated using the bootstrap resampling technique, showing an impressive ability to differentiate and establish accuracy. Moreover, the nomogram provides a reliable and precise assessment of survival duration for patients with T1–T2 SGSCC, aiding in the design of individualized treatment plans.

The prognostic nomogram demonstrates good predictive power and clinical utility in determining OS and CSS for patients with T1–T2 SGSCC. Therefore, it can be considered an essential tool in determining the treatment plan for patients diagnosed with T1–T2 SGSCC.

## Data Availability

The public database data used in the current study analysis is available in the SEER repository (https://seer.cancer.gov/). Validation set 2 is available from the corresponding author upon reasonable request.
